# Sex Specific Determinants in Osteoarthritis: A Systematic Review of Preclinical Studies

**DOI:** 10.3390/ijms21103696

**Published:** 2020-05-24

**Authors:** Deyanira Contartese, Matilde Tschon, Monica De Mattei, Milena Fini

**Affiliations:** 1 Laboratory of Preclinical and Surgical Studies, Rizzoli RIT Department, IRCCS–Istituto Ortopedico Rizzoli, 40136 Bologna, Italy; deyanira.contartese@ior.it (D.C.); milena.fini@ior.it (M.F.); 2 Department of Medical Sciences, University of Ferrara, 44121 Ferrara, Italy; monica.demattei@unife.it

**Keywords:** osteoarthritis, gender, sex, molecular mechanisms, gender medicine, preclinical model

## Abstract

Osteoarthritis (OA) is a highly prevalent joint disease that primarily affects about 10% of the world’s population over 60 years old. The purpose of this study is to systematically review the preclinical studies regarding sex differences in OA, with particular attention to the molecular aspect and gene expression, but also to the histopathological aspects. Three databases (PubMed, Scopus, and Web of Knowledge) were screened for eligible studies. In vitro and in vivo papers written in English, published in the last 11 years (2009–2020) were eligible. Participants were preclinical studies, including cell cultures and animal models of OA, evaluating sex differences. Independent extraction of articles and quality assessments were performed by two authors using predefined data fields and specific tools (Animals in Research Reporting In Vivo Experiments (ARRIVE) guideline and Systematic Review Centre for Laboratory animal Experimentation (SYRCLE) tool). Twenty-three studies were included in the review: 4 in vitro studies, 18 in vivo studies, and 1 both in vitro and in vivo study. From in vitro works, sex differences were found in the gene expression of inflammatory molecules, hormonal receptors, and in responsiveness to hormonal stimulation. In vivo research showed a great heterogeneity of animal models mainly focused on the histopathological aspects rather than on the analysis of sex-related molecular mechanisms. This review highlights that many gaps in knowledge still exist; improvementsin the selection and reporting of animal models, the use of advanced in vitro models, and multiomics analyses might contribute to developing a personalized gender-based medicine.

## 1. Introduction

Sex has an important role in the pathophysiology of several diseases, including osteoarthritis (OA). OA is a highly prevalent joint disease that primarily affects the elderly population causing frailty in patients [1,2]. The World Health Organization (WHO) Scientific Group on Rheumatic Diseases estimated that 10% of the world’s population who are 60 years or older present significant clinical problems attributed to OA [3]. Although OA may affect any joint in the body, it most commonly affects the hips, knees, hands, and spine, causing musculoskeletal disability and resulting in limited activities of daily living in the adult population, reduced mobility, and increased levels of healthcare utilization [4,5,6,7]. OA is defined as a multifactorial condition of joint failure mainly characterized by articular cartilage loss, subchondral bone sclerosis, and inflammation, leading to progressive joint degradation, structural alterations, loss of mobility and function, pain, and decreased quality of life [8,9,10]. The risk factors associated with OA are multiple and can be genetic, metabolic, biochemical and biomechanical in nature, and patient related factors, including age, gender, obesity, and hormone levels, thus contributing to the unbalance of the delicate equilibrium between anabolic and catabolic activities of chondrocytes [11]. For example, an inappropriate mechanical joint stress damages the integrity of the extracellular matrix and influences the chondrocytes metabolism, thus reducing their anabolic ability to replace basic/healthy matrix components, increasing the release of pro-inflammatory cytokines (interleukin (IL)-1β, IL6, IL8, and tumor necrosis factor (TNF)-α), which in turn increase the expression of catabolic factors (metalloproteinases (MMPs), nitric oxide (NO), prostaglandin E2 (PGE2) and cyclooxygenase 2 (COX-2)) and stimulate apoptosis and necrosis [12,13,14]. Structural changes visible on radiography include joint space narrowing, osteophyte formation, synovial inflammation and fibrosis, pathological changes in ligaments, menisci and tendons, and bone remodeling [15,16]. Despite being poorly studied, gender represents one important risk factors in the development of OA [17]. The incidence of knee, hip, and hand OA is higher in women than men, and it increases dramatically in women around the time of menopause [18,19]. Therefore, some authors have suspected a role of hormonal factors in the development of OA [20]. Estrogens have been reported as protective for OA in some studies and linked to the onset of OA in others, indicating they have many complex effects that may affect the onset of the pathology as well as its evolution [21,22]. However, data are conflicting [23], since differences between men and women could be explained not only by hormonal variations but also by other factors (reduced volume and thickness of cartilage, bone loss or lack of muscle strength). The knowledge of sex differences in OA pathophysiology would be of key importance because of population aging and health systems must prepare for the large increase in the number of people with OA requiring health services. For this reason, there is the need to better understand the mechanisms of the pathology and potential sex related differences through adequate preclinical models, to evaluate new hypotheses, concepts, and therapeutic treatments capable of arresting or reverting disease progression [14,24].

The rationale of the study was to deeply investigate the current state of knowledge and point toward implementation of knowledge gaps.

The purpose of this study was to systematically review the preclinical studies, involving both in vitro and in vivo papers, of OA models investigating sex differences in OA, with particular attention to the molecular aspect and gene expression, but also to the histopathological aspects.

## 2. Results

**Figure 1:**
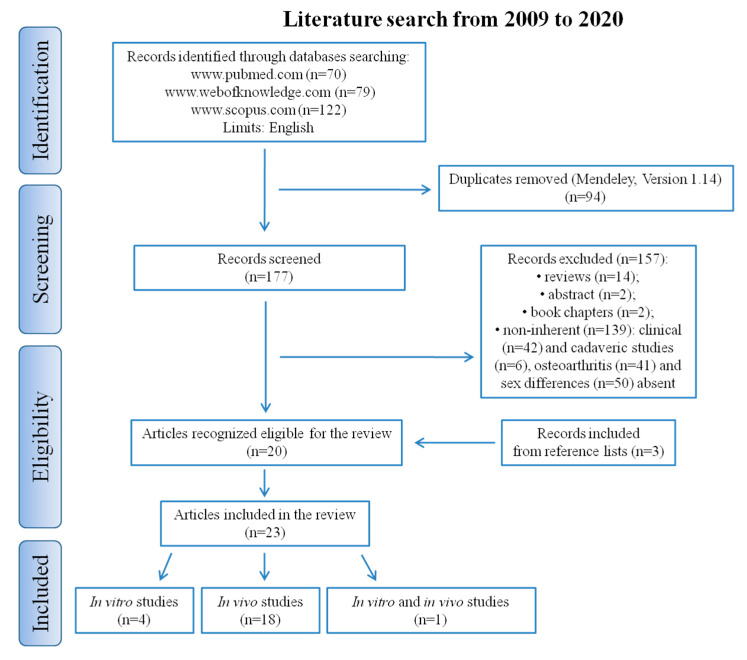
Search strategy according to Preferred Reporting Items for Systematic Reviews and Meta-Analyses (PRISMA) guidelines.

An initial literature search was performed using the previously mentioned keywords: 70 articles were retrieved using Pubmed www.pubmed.com, 122 articles using Scopus www.scopus.com, and 79 articles using Web of Knowledge www.webofknowledge.com. Subsequently, the resulting references were submitted to a public reference manager (Mendeley 1.14, “www.mendeley.com”) to eliminate duplicate articles (*n* = 94). The remaining papers (*n* = 177) were screened for matching with the inclusion criteria. Reviews (*n* = 14), abstracts (*n* = 2), book chapters (*n* = 2), and non-inherent papers including clinical (*n* = 42) or cadaveric (*n* = 6) studies, papers with no osteoarthritis (*n* = 41) or with no sex differences (*n* = 50) were excluded. After screening, a total of 20 articles were recognized as eligible for the review and examining the reference lists of these studies, another three papers were included. A total of 23 studies were definitely included in the review: 4 articles were in vitro studies, 18 in vivo, and 1 both in vitro and in vivo (Figure 1).

### 2.1. Preclinical in Vitro Studies

The in vitro studies were analyzed based on the cell source (Table 1).

Most in vitro studies were performed by isolating cells from human tissues (3 papers out of 5; 60%), then, in the same way, by canine tissues (1 paper out of 5; 20%) and rats (1 out of 5; 20%).

#### 2.1.1. Human Tissues

The majority of studies (*n* = 3) used human cells isolated from different tissues of male and female patients affected by OA and subjected to total knee arthroplasty (TKA) [25,26,27].

Overall, these three studies analyzed samples from 400 patients, of which 146 were males and 254 females (64% female prevalence). Retrieved tissues were used both for cell isolation and for a wide plethora of assays (histological, immunohistochemistry (IHC), immunoblotting, microarray, fluorescent in situ hybridization (FISH), gene expression, and ELISA analyses). The works of Koelling and Pan were well structured with a clearly reported diagnosis of OA (Kellgren–Lawrence grading scale or the American College of Rheumatology (ACR) classification) [25,26]; while the work of Stumm, besides having the lowest number of patients, did not give evidence of OA diagnosis [27]. All studies harvested control tissues or from minimally eroded surfaces or from healthy donors, thus including a control, not OA; moreover, they specified that chondrocytes used for experiments were at passage one or cultured in three-dimensional (3D) culture conditions in order to retain the chondrocyte phenotype.

Sex differences were observed in synovial fluid composition: males had increased levels of testosterone, MMPs, sulphated glycosaminoglycans (sGAGs), transforming growth factor (TGF)-β1, TGF-β2, and hepatocyte growth factor (HGF), whereas females had higher levels of macrophage stimulators (leukemia inhibitory factor (LIF), macrophage colony-stimulating factor (M-CSF), macrophage migration inhibitory factor (MIF)), pro-inflammatory mediators (growth-regulated oncogene α (GRO-α), monocyte chemotactic protein-3 (MCP-3), and monokine induced by gamma interferon (MIG)), inflammatory interleukins (IL2α, IL3, IL12p40, IL16, IL18, and TNF-β), TNF-α, and TNF-related apoptosis-inducing ligand (TRAIL) [25,26].

In chondroblast and chondrocyte cultures, female OA cells had lower mRNA expression of vitamin D_3_ receptors (VDRs) and of protein disulfide isomerase A3 (PDIA3), and higher expression of estrogen receptors (ERs) compared to male cells. Female cells from minimally eroded surfaces had less VDR mRNA than male cells but comparable levels of mRNAs for the other three receptors, and higher mRNA levels of pro-inflammatory interleukins (IL1A, IL6, and IL8) in comparison to male cells. Both OA and non OA female cells had reduced expression of signaling molecules (WNT5A and DKK2) than male cells [26]. Moreover, Koelling analyzed by microarray the expression activities of many genes and found that 4.9% of genes (ESR1, ESR2, AR, Sox9, Runx2, Col I, and Col II) were differently expressed between the sexes [25]. Cells differed also in their responsiveness to sexual hormones: in general, cultures from females exerted responsiveness to estrogens and males to androgens and vitamin D_3_. Sexual hormones differently regulate the chondrogenic gene expression in a sex-specific and dose-dependent manner; their actions are also modulated by an upregulation and downregulation of receptors. In a study by Stummet al., the authors found an age dependent effect on loss of the Y-chromosome in chondrocyte cultures from male donors, but no sex differences in gene expression between cells from male and female donors; although their samples size (eight patients) was low and limited [27].

In osteoblast cultures, ER expression was increased in female cells in comparison to male cells, while VDR expression was increased in males. Treatment with 17β-estradiol (E2) induced an increase in alkaline phosphatase (ALP) and osteocalcin (OC) and a decrease in osteoprotegerin (OP) in females compared to males, while treatment with 1α,25(OH)2D3 induced an increase in ALP in males compared to females. 

#### 2.1.2. Canine Tissues

One study used osteoblasts isolated from subchondral, trabecular, and cortical bone from the femoral head of male and female dogs undergoing total hip replacement (THR) (36% female prevalence) [28]. Cells cultured in mineralizing medium were evaluated for cell viability and number, mineralization, and tissue non-specific alkaline phosphatase (TNAP) activity. Results showed no differences in any of the analysis parameters irrespective of the bone tissue types between males and females. Asonly one study, the results lack a suitable evidence-based level.

#### 2.1.3. Rats

One study used fibroblast-like synoviocyte (FLS) isolated from the synovial membrane of female and male Sprague-Dawley rats (*n* =16 animals, 50% female prevalence) with temporomandibular joint (TMJ) OA chemically induced by Freund’s adjuvant combined with monosodium iodoacetate (MIA) [29]. Cells cultured in a co-culture system with a macrophage cell line (NR8383) were treated with or without TNF-α, and evaluated through ELISA, Western blot, and RT-PCR. Results showed increasedinducible nitric oxide synthase (iNOS), IL-1β, and monocyte chemoattractant protein-1 (MCP-1) expression, and an increase in macrophages number and migration in female cells compared to males in the presence of TNF-α stimulation, while no difference without treatment was observed.

### 2.2. Preclinical in Vivo Studies

#### 2.2.1. Quality and Risk of Bias Assessments

**Figure 2:**
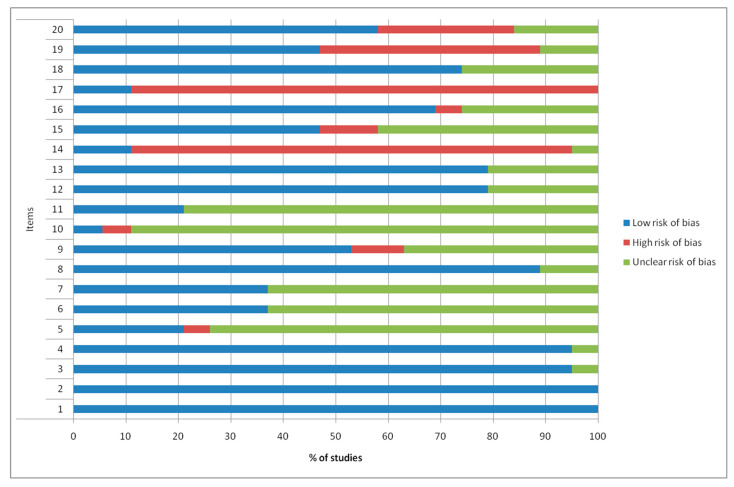
Frequencies (%) of quality assessment according to Animal Research Reporting In Vivo Experiment (ARRIVE) criteria.

**Figure 3:**
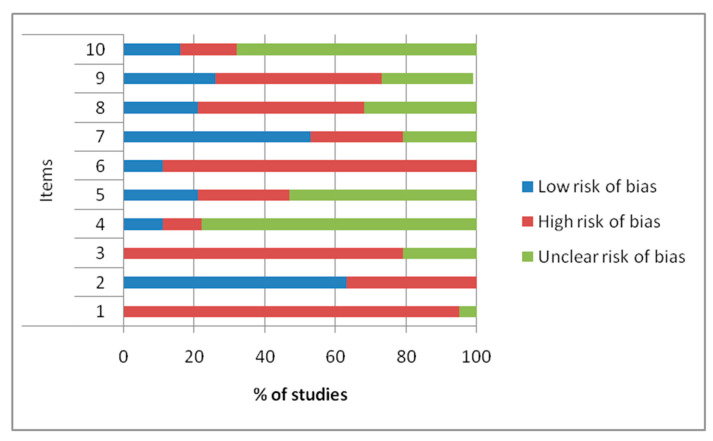
Frequencies (%) of risk of bias assessment according to Systematic Review Centre for Laboratory animal Experimentation (SYRCLE) tool.

Quality and risk of bias assessments are shown in Figure 2 and Figure 3.

The results of Animals in Research Reporting In Vivo Experiments (ARRIVE) showed that higher risk of bias was observed in most papers at items 14 “Results/baseline date” and 17 “Results/adverse events”, with frequencies of 84% and 89%, respectively. Items 1 “Title”, 2 “Abstract”, 3 “Introduction/background”, 4 “Introduction/objectives”, 8 “Methods/experimental animals”, 12 “Methods/experimental outcomes”, 13 “Methods/statistical methods”, 16 “Results/outcomes and estimation”, 18 “Discussion/interpretation, scientific implications”, and 20 “Discussion/funding” presented low risk of bias, with 100%, 100%, 95%, 95%, 89%, 79%, 79%, 69%, 74%, and 58% of frequencies, respectively. Remaining items (5 “Methods/ethical statement”, 6 “Methods/study design”, 7 “Methods/experimental procedures”, 9 “Methods/housing and husbandry”, 10 “Methods/sample size”, 11 “Methods/allocating animals to experimental groups”, 15 “Results/numbers analyzed”, and 19 “Discussion/generalizability, translation”) presented an unclear risk of bias.

The results of the Systematic Review Centre for Laboratory animal Experimentation (SYRCLE) showed that higher risk of bias was observed in most papers at the items 1 “allocation bias”, 3 “allocation concealment”, and 6 “random selection for outcome assessment”, with frequencies of 95%, 79%, and 89%, respectively. Items 2 “baseline characteristic” and 7 “blinding of outcome assessor” presented low risk of bias, with frequencies of 63% and 53%, respectively. The remaining items (4 “random housing of animals”, 5 “blinding of caregivers and investigators”, 8 “incomplete outcome data”, 9 “selective outcome reporting”, and 10 “other bias”) presented a low-to-unclear risk of bias.

The in vivo studies were evaluated based on the animal species (Table 2).

Most in vivo studies were performed in mice (11 out of 19;57.9%), followed by rats (4 out of 19;21.1%), pigs/mini-pigs (2 out of 19;10.6%), guinea pigs (1 out of 19;5.3%), and baboons (1 out of 19;5.3%).

#### 2.2.2. Mice

The majority of studies (*n* = 11) used the mouse as an animal model to evaluate the development of OA [30,31,32,33,34,35,36,37,38,39,40].

Several models are used for mimicking OA in mice: spontaneous models of OA, which include naturally occurring OA [33,39] and use of genetic modifications [30,35,38]; surgically induced models of OA through technical meniscectomy (MMT) or destabilization of the medial meniscus (DMM) [30,32,36,37,40]; metabolically induced models of OA by high-caloric diets [31,34].

In two studies STR/Ort mice were used as spontaneous OA models naturally occurring: both authors observed that differences in bone structure between female and male mice may explain the OA development in this strain [33,39] with an age-related decline of bone marrow space in both males and females, adecrease in cancellous bone mineral density (BMD) in males compared to females, and an higher trabecular bone mass and greater cross-sectional of cortical bone in females than males [33,39].

By resorting to genetic modifications to induce OA, one study used mice lacking frizzled-related protein (Frzb), a gene associated to OA, to study its effect as well as the addition of voluntary running wheel exercise performance and OA development [35]. At 6 months, both female and male Frzb−/− mice showed reduced exercise performance compared tothe control. However, running exercise did not significantly affect severity of OA either in males or females.

Another study used mice with a mutation of matricellular protein NOV (nephroblastoma overexpressed) (NOV^del3^), commonly implicated in the maintenance of joint homeostasis, to determine if its disruption causes joint degeneration [38]. Results showed that compared to females, males exhibited severe OA-like pathology at 12 months (with high Osteoarthritis Research Society International (OARSI) score), affecting all tissues of the joint. The expression of collagen I (Col I), collagen X (Col X), and proliferating cell nuclear antigen (PCNA) in male mice was greater compared to females. The density of articular cartilage cells of control mice is maintained at a constant level from 2 to 12 months of age, whereas this is not the case in Nov^del3^−/− mice, in which a significant increase in cell density was observed in males at 2 months, and a decrease at 6 and 12 months in both males and females. Therefore, NOV is necessary for maintenance of articular cartilage and for joint homeostasis, its disruption causes OA-like disease in Nov^del3^−/− mice, with greater severity in males compared to females.

One study used Atg5cKO mice lacking the autophagy-indispensable Atg5 gene in their chondrocytes, to analyze the development of spontaneous OA, both upon aging and post-traumatic surgically induced (partial MMT) [30]. With aging, results showed a development of OA particularly severe, with cell death of articular chondrocytes, in males compared to females. Upon surgery, males developed a more severe OA compared to females, however, in both sexesno differences were seen between the control and Atg5cKO animals. Therefore, autophagy protects articular cartilage and the abrogation of this process leads to the development of OA upon aging, especially in male mice, while it has no effect in the development of post-traumatic OA.

One study used C57BL/6J mice to investigate sex effects on pain-related behaviors and knee damage following MMT [40]. Results showed more severe cartilage damage in males than in females, butcomparable results between sexes in osteophyte formation. In addition, both sexes developed comparable heat hypersensitivity in the acute phase, although this was delayed in females compared to males. Locomotor activity was higher in females compared to males in the acute phase, indicating that females were more active at the beginning of the test, regardless of the knee condition.

Two studies used mice with a C57BL/6J background: wild-type, CD-1, and ADAMTS-5 (a disintegrin and metalloproteinase with thrombospondin motifs), the latter genetically modified lacking the Adamts5 gene responsible for damage to joint tissues, to evaluate OA development and mechanical allodynia, following DMM [32,36]. Results showed severe OA and mechanical allodynia in male wild-type mice compared to females, with thicker subchondral bone plates, cartilage degradation and bone volume fraction higher in old than young. In CD-1 mice, both sexes developed OA changes with concurrent mechanical allodynia; while both male and female ADAMTS-5 mice did not develop OA changes or mechanical allodynia. Therefore, these genetically modified mice are resistant to DMM-induced OA-like lesions and to the associated mechanical allodynia. Finally, one study used wild-type mice and integrin α1-deficient BALB/c mice to determine in serum metabolic changes and OA development following DMM surgery and treatment with the epidermal growth factor receptor (EGFR) inhibitor erlotinib [37]. Erlotinib treatment resulted in a decrease of multiple OA signs in the female mice including bone volume and density, subchondral bone thickness, and cartilage degradation, but not in males. Therefore, the effects of erlotinib treatment were ‘on target’ and sex dependent.

Moreover, two studies, always using genetically modified mice, evaluated metabolic effects of high-caloric diets on inflammatory processes and OA development [31,34]. Results from the first study showed that reduction into the ratio of polyunsaturated fatty acids (PUFAs) caused a modest decrease in the inflammatory cytokine IL6 both in males and females (52% vs. 46%), while TNF-α decreased to a lesser extent in male mice compared to female mice (19% vs. 41%). However, these systemic changes did not reduce osteophyte development, synovial hyperplasia, or cartilage degeneration [31]. In a second study, transgenic hCRP, LDLr-/-, and ApoE*3Leiden.CETP mice based on C57BL/6J background were used [34]. Results showed that long-term feeding of high-caloric diets showed significant OA aggravation upon feeding, whereas females gained less weight and did not develop diet-induced OA. ApoE*3Leiden.CETP mice rapidly developed obesity and were highly prone to cartilage degradation. Specifically, in males, OA scores were consistently higher than in females. In addition, ApoE*3Leiden.CETP males showed high scores for both osteophyte formation and synovitis, while females developed almost no osteophytes but demonstrated high synovitis scores and significantly higher plasma cholesterol levels. Therefore, diet-induced metabolic dysfunction per se does not necessarily lead to aggravated articular cartilage degradation and to OA development in mice on C57BL/6J background.

#### 2.2.3. Rats

Different studies (*n* = 4) used rats as animal species (Sprague-Dawley and Fischer strains), with an age range from 8 weeks to 24 months, to experimentally create a reliable OA model [29,41,42,43].

In the majority of studies (*n* = 3), OA was chemically induced by an injection of MIA or MIA combined with complete Freund’s adjuvant (CFA) in the knee or TMJ [29,41,42].

In the remaining study, OA was surgically induced in the TMJ through the experimental creation of malocclusion [43].

Overall, in a range from 2 to 4 weeks, results showed larger OA-like degenerative regions in female rats compared to male rats, with cartilage loss and thinning, reduced bone volume, subchondral bone deterioration, osteophyte formation, chondrocytes loss, and synovial inflammation. In addition, from a molecular point of view, the gene expression and immunohistochemical signals of insulin-like growth factor-1 (IGF1), its type 1 receptor (IGFR1), IGF binding protein-3 (IGFBP3), extracellular protein, and matrix proteins, mostly Col II, were reduced in the female group compared to the male group, consistent with the observation of greater OA severity, cartilage damage, and disrupted reparative response in females [43]. Moreover, increased IHC staining and mRNA expression of iNOS, IL-1β, MCP-1, and greater amounts of CD68-positive macrophages infiltration were detected in the synovial membrane of females compared to that of males. These results indicate that the female synovial membrane is responsible for secreting greater amounts of pro-inflammatory factors, showing more severe synovitis, with hyperplastic synovial lining, as compared to males. Another important aspect considered by two of the mentioned studies was the sex and age differences in response to pain and tactility [41,42]. Results clearly demonstrated that older rats exhibited significantly more OA-like pain and a more pronounced and longer-lasting hyperalgesia than young rats; and female rats showed greater pain responses, higher susceptibility, and central sensitization as compared to males. Therefore, these observations indicate that females represent the most vulnerable group to OA and to related pain and inflammation conditions.

#### 2.2.4. Guinea Pigs

Only one study used the guinea pig as an animal model (male and female Hartley, and female strain 13) to examine sexdifferences in the development of spontaneous knee OA [44]. Cartilage degeneration and subchondral BMD and meniscal mineral density (MD) in guinea pigs 12 months of age were examined. Results showed that male Hartley guinea pigs and female strain 13 guinea pigs had more severe cartilage degeneration and higher subchondral BMD than female Hartley guinea pigs. Meniscal MD in female Hartley guinea pigs and in female strain 13 guinea pigs was lower than the meniscal MD in the male Hartley guinea pigs. Finally, no significant differences were found for meniscal calcium content between males and females. In conclusion, the study demonstrated that higher subchondral BMD, but not meniscal MD, is associated with more severe cartilage degeneration in guinea pigs, suggesting that subchondral bone metabolisms may play a role in the development and/or progression of OA. However, being the only study performed in guinea pigs, no relevant evidences on sex differences can be extrapolated for this kind of model and OA. 

#### 2.2.5. Pigs/Mini-Pigs

In two 2015 studies, Kiapour et al. employed the pig (Yorkshire) or mini-pig (Yucatan) animal model to evaluate the role of sex on biomechanical outcomes in a model of surgically induced OA through the anterior cruciate ligament (ACL) transaction and repair [45,46]. Female and male animals underwent ACL reconstruction using conventional (bone-patellar tendon-bone graft) or bio-enhanced allografts with an extracellular matrix (ECM)-based scaffold and autologous platelet-rich plasma (PRP) (with absorbable or nonabsorbable sutures). In both studies, ACL structural, biomechanical, and histological properties, knee laxity, and cartilage damage were assessed after 15 weeks. Female pigs treated with bio-enhanced allografts and with absorbable sutures had poorer results from a biomechanical point of view with a lower linear stiffness, yield, and maximum load, and a greater knee laxity as compared to males. In addition, ACL grafts in males showed significantly greater vascularization when compared to females, while other histological parameters (cellular and collagen contents) showed no sex-related differences. Lastly, female pigs had a significantly larger area of cartilage damage after conventional ACL reconstruction than their male counterparts. This difference was not seen for the groups treated with bio-enhanced allografts, in particular with ECM-based scaffold and PRP, suggesting a greater protective effect of the bio-enhancement on the cartilage of the female knees. In conclusion, these studies showed that female pigs had significantly poorer biomechanical outcomes compared to males after 15 weeks of healing, when repaired with an absorbable suture. Finally, no significant differences were found for weight and range of motion (from pre-injury to post-injury) between males and females.

#### 2.2.6. Baboons

One study used the baboon (*Papiohamadryas*) as an animal model to investigate sex-specific variation and effect of age on occurrence and severity of spontaneous knee OA in animals aged from 4.5 to 33 years [47]. By analyzing the joint of female and male baboons, an OA severity macroscopic score was assigned, recording articular cartilage degradation and presence/absence of osteophytes. Data showed a spontaneous OA more frequent and severe in older animals; males developed OA earlier with cartilage degeneration, while females progressed more quickly to advanced disease. However, as the only study performed in baboons, no relevant evidences on sex differences can be extrapolated for this kind of model and OA.

## 3. Discussion

Osteoarthritis is a serious pathology that causes persistent morbidity such as pain, fatigue, and disability, and has an enormous burden on a person’s day-to-day quality of life [2]. Currently available treatments consist of analgesics, opioids, glucocorticoids, non-steroidal anti-inflammatory drugs (NSAIDs), viscosupplementation, physical exercise, diet, and surgery in the final stages of the disease [48,49]. However, these strategies are able to delay but not stop the progression of OA, with pain and inflammation relief and symptoms improvement. The OA pathophysiological mechanisms are difficult to understand because of the complexity of the disease in terms of quantity and the diversity of risk factors and tissues involved [10]. 

For years, OA was considered as an age-related pathology, but during the past decade, it has been observed that sex represents a well-known variable that influences its development. The lack of knowledge related to the sex differences in OA pathophysiology, especially at the molecular level, is partly due to the deficiency in studies that evaluate and examine this aspect, but also to the lack of relevant preclinical models. In this context, the present a review aimed to describe the results of in vitro and in vivo studies taking into consideration the gender balance and the related differences between sexes in the field of OA to highlight their possible implications in therapeutic approaches. Taking into account the numbers, in vitro studies analyzed 426 samples in total, of which 161 were retrieved from male and 266 from female subjects (62% female prevalence, independently from the type of tissue and human or animal donors); in vivo studies performed in mice used 458 animals of which 232 were male and 226 female (51% male prevalence), in rats 294 animals of which 153 were male and 141 female (52% male prevalence), in pigs/mini-pigs 58 animals of which 31 were male and 27 female (53% male prevalence), and in guinea pigs 15 animals of which 5 were male and 15 were female (75% female prevalence). Studies using baboons were gender balanced using 153 subjects. In total, the retrieved studies analyzed 1563 samples of which 735 were male and 828 were female (overall 53% female prevalence). These numbers, apart those in vitro in which samples from female subjects are prevalent, reflect the quite homogeneous distribution of sex (550 males vs. 562 females). However, preclinical studies testing on both sexes remain the minority of available studies in the literature.

In the in vitro studies, human or animal derived samples were used for histology, immunohistochemical analyses, growth factor, hormone measurements, and cell isolation, with the chondrocyte phenotype prevalently investigated. Apart from the scarce number of studies retrieved on this topic, sex differences regarded the molecular composition of synovial fluid, as highlighted in particular by the work of Pan [26]; males expressed more catabolic enzymes, such as MMPs, but also expressed many anabolic growth factors and sGAGs, while females had higher values of inflammatory cytokines that could explain the higher pain they experienced. This finding was confirmed in the work of Xue on rats [29]: they found greater pro-inflammatory cytokine production and gene expression (particularly, iNOS and IL-1β) in female compared to male synoviocytes, together with an increase of recruited macrophages and related MCP-1 production. These papers suppose that estrogens have an impact on an increased sensitivity to inflammatory stimuli and reactions in female, and that males have better compensatory and anabolic pathways. However, in these studies, estrogen evaluations were not performed. As far as cell cultures (chondrocytes and osteoblasts) were concerned, they differed both in terms of expression of hormone receptors and consequently in their responsiveness to hormonal stimulation. Generally, physiologic concentrations of estrogen help the regenerative potential of cartilage in females but not in males, while testosterone does it in males and not in females [25]. This suggests a sexual dimorphism in the development and regeneration mechanisms in OA. Although, a possible conversion of testosterone into estradiol, via the aromatase pathway, is presumed, thus, the regenerative effects of aromatase inhibitors (particularly in men) should be deeply investigated. Moreover, chondrocytes isolated from 20 OA patients exhibited sex differences in the expression of many genes: inflammatory cytokines, enzymes involved in matrix degradation, and WNT signaling molecules [26]. It should be noted that, when samples were retrieved from a smaller number of subjects, as in the work of Stumm or Meeson [27,28], no statistical evidence could be highlighted between sexes. So far, to highlight statistically significant differences related to sex, a large number of patients, samples, and cell culture replicates should be considered. From in vitro studies using samples from animal tissues, no clear conclusion can be drawn because of the lack of evidence due to the scarce number of utilized samples.

Benefits of the in vitro studies are the ease of performing cultures that in turn allow to study many aspects, from basal expression of phenotypic markers to gene expression and secretion of cartilage matrix molecules or pro-inflammatory mediators or DNA and sex chromosome differences, both as their intrinsic properties and in responses to hormonal fluctuation or stimulation. Moreover, the possibility of isolating cells from pathological OA tissues is of relevance to in vitro set up features typically expressed in OA pathology in order to improve the OA basic knowledge and to test regenerative/reparative therapeutic innovative strategies.

The drawbacks of in vitro studies are the possibility of cell transformation or dedifferentiation, the lack of microenvironment and surrounding matrix and, *in primis*, when dealing with pathological cultures, the difficulties in obtaining samples from healthy donors to have comparable controls. Many authors retrieved samples from non-loading areas or areas not affected by OA; however, it should be emphasized that they were harvested from a chronic inflamed microenvironment [25,26]. 

However, when testing new therapeutic approaches (i.e., biomaterials, cells, growth factors, drugs, biophysical therapies, and medical devices or their associations) for the treatment of OA, in vivo models cannot be ignored.

In the in vivo studies analyzed in this review, to evaluate the sex differences in OA development, the most commonly used animal model was the mouse. This model is a primary choice because of low maintenance costs, the progress in mice genetics, and easy availability, which allow the evaluation of changes in OA joints and allow the possibility to induce several genetic modifications [50]. However, other animals, such as rats, pigs/mini-pigs, guinea pigs, and baboons were also employed; the great heterogeneity of studies makes the comparison difficult. In addition to the animal species, another aspect to be considered is the choice of the model for OA induction.

In half of the studies included in this review, spontaneous models of OA were used, including naturally occurring osteoarthritis (22%), genetic modifications (16%), and metabolically induced models by high-caloric diets (11%); the other half of the studies comprised the surgical induction of OA by MMT, DMM or ACL (42%) and chemical induction (16%) by an injection of MIA in the joint. Knee OA was prevalently studied (84%) followed by TMJ (16%). Macroscopic and histological scores, X-ray, micro-computed tomography (μCT), biomechanical testing, and immunohistochemical and histological analyses were performed.

Main sex differences regarded the OA development and severity in terms of bone architecture, osteophytes formation, synovial inflammation, cartilage degeneration, BMD, subchondral bone deterioration, and pain development. In particular, female rats showed a more severe synovial inflammation, cartilage destruction, and subchondral bone deterioration than males. Females were more sensitive to inflammatory stimulus and showed more extensive CD68-positive macrophage infiltration around the synovial membrane [29,43]. This may explain the higher pain in females compared to males [41,42]. In addition, molecular investigations show that the expression of IGF1, IGFR1, and Col II is reduced in females, consistent with the observation of greater OA severity compared to males [29,43]. Sex differences were also reported in a large animal model as pig/mini-pig. In this case, females showed worse biomechanical outcomes (i.e., stiffness, yield, and maximum load), weaker and less vascular grafts, more lax knees, and greater cartilage damage than males following ACL repair; these factors, however, improved with the addition of an ECM-based scaffold loaded with autologous PRP [45,46]. Naturally-occurring knee OA is common in mice, guinea pigs, and baboons. Despite the scarce number of papers retrieved on these models, sex differences in OA development highlighted that male baboons begin to show earlier and mild articular cartilage degeneration, while females progress more rapidly to advanced stages of disease [4]. Contrarily, for guinea pig and mouse models an inverted situation was observed, with bone architectural changes (for example, greater tibia curvature and ellipticity), more severe cartilage degeneration, and higher subchondral BMD in males compared to females [33,39,44]. This association between subchondral and cartilage changes suggests that subchondral bone metabolism could play a role in the development and/or progression of OA, affecting the biomechanical properties of the bone, and the release of cytokines and growth factors, which may promote cartilage destruction. Finally, also following surgical induction of OA, greater damages with higher allodynia and subchondral bone plate thickening in male than female mice were observed [32,36,40]. Using genetic modifications in mice, such as deletion of the Frzb and Atg5 gene, or Nov targeted mutation, a depletion of articular cartilage in cell density was observed, as well as an increase in collagen fiber thickness and more Col I and Col X positive cells in the damaged areas, which indicate abnormal differentiation of chondrocytes with a more evident effect in males than females [30,35,38]. Thus, specific genes are essential for protecting and maintaining the morphology and cell density of articular cartilage; their abrogation leads to the development of OA, especially in male mice. However, when an animal model is adopted, it should be taken into account that sex affects OA development; it is well known that in male mice spontaneous and induced OA occurs more frequently than in female mice, probably due to sexual hormonal variations, although their mechanisms remain unclear [24]. 

From this review it could be concluded that from the in vitro viewpoint there is a lack of representative and advanced 3D in vitro models of OA investigated to give evidences of sex and gender related differences. In the field of OA, many advanced in vitro models exist to study this pathology: they comprise direct and indirect bi- and tri-cultures, by cultivating two or three different cell phenotypes, 3D cell cultures, as chondrocyte micromasses cultured in the presence of an external pathological stimulus as IL1-β, TNF-α or synovial fluid from OA patients, or tissue and organ culture, as cartilage or osteochondral explant culture. These models have numerous advantages in comparison with two-dimensional (2D) or monolayer cultures, such as the retention of ECM and intrinsic growth factors, the possibility to extend culture experimental times up to many weeks, and the possibility to perform histology and biomechanics on samples [51].

Moreover, when we talk of sexual hormones and their actions in contributing to sex-related differences in OA, it should be empathized that many factors should be considered as relevant: (i) circulating levels of sex hormones, (ii) local production, and (iii) presence and functionality of receptors and related signaling pathways [19]. In some studies, the gene expression or protein levels of receptors have been evaluated but in the absence of their functional assays; so far, the detection and quantification of a molecule (at both protein or gene expression levels) should be coupled with functional tests or inhibition tests. 

Of main importance, by setting robust and representative in vitro models, the reduction and/or replacement of animal use could be pursued, thus being compliant with ethical principles of the three R’s of Russell and Burch (reduction, refinement, and replacement) and legislative decree (Directive 2010/63/EU). However, in this field, animal models remain irreplaceable as alternative models since in vitro, in silico, and robotic models cannot recreate the complete biological setting of an organism particularly in a trauma or pathology.

From the in vivo viewpoint, there is no “gold standard” animal model used in OA and sex related researches, but several models aim to reproduce processes and OA lesions [24,52]. Most of the studies analyzed in this review used small animals such as mice, rats, and guinea pigs, due to cost-effectiveness, ease of handling and housing, and opportunity for genetic manipulations. No studies have used medium and large animals such as rabbits, sheep, goat, orhorses [50,53]. These models allowed to identify sex differences, mainly in the histopathological aspects, showing a higher prevalence of OA and greater bone and cartilage architecture changes in males compared to females, and particularly in mice, regardless of the OA induction method. However, in contrary to in vitro works, the analysis of sex-related molecular mechanisms isstill lacking. The evident heterogeneity of the retrieved papers demonstrated the lack of consistency and evidence-based results that could help in understanding sex differences in the etiology, molecular events, and signaling pathways which ultimate in OA development. On the other hand, this is not surprising because of the limits in the identification of an appropriate animal model, especially in complex diseases, such as OA [54]. Since differences in type of model, animal species, and OA induction methods can lead to different experimental outcomes, there is the need to reduce experimental variability and to increase the reliability of data by boosting sample sizes, adopting in vitro advanced models, and revealing basic and molecular aspects involved, in particular in the era of omics, that can lead to a better understanding of how cellular processes are differently regulated in males and females.

## 4. Materials and Methods 

### 4.1. Search Strategy

The present systematic literature review involved a systematic search carried out according to the Preferred Reporting Items for Systematic Reviews and Meta-Analyses (PRISMA) statement in three electronic databases (PubMed, Scopus, and Web of Knowledge www.pubmed.com, www.scopus.com and www.webofknowledge.com). The search was applied with the following keywords: “osteoarthritis AND (gender OR sex) AND (in vitro model OR in vitro culture OR in vivo model OR animal model OR preclinical model)”. The screening process and analysis were conducted separately by two independent observers (D.C. and M.T.). Firstly, the articles were screened by title and abstract, using the following inclusion criteria: in vitro and in vivo reports about gender differences in osteoarthritis models, full text, written in English language, published in the last 11 years (2009–2020). Exclusion criteria were articles written in other languages, reviews, abstracts, book chapters, clinical and cadaver studies, reports in which the model of osteoarthritis was absent and articles in which gender differences were not taken into consideration. Secondly, the reference lists of the included papers were screened to obtain further studies. Included papers were grouped according to the test model used, whether in vitro, in vivo or both. Thirdly, duplicates were removed. Disagreements were resolved by discussion and where resolution was not possible, a third reviewer was consulted (M.F.).

### 4.2. Data Extraction

General characteristics were extracted by D.C. and M.T., including details of the study (the first author’s name and year of publication) as well as main findings. The data were extracted into a structured data collection form by one reviewer (D.C.) and checked for accuracy and completeness by a second one (M.T.). From each article dealing with an animal model, the following information was extracted: animal number and strain (age), osteoarthritis model, aim, and follow-up evaluations. From each article dealing with an in vitro model the following information wasextracted: cell source and phenotype, osteoarthritis diagnosis, culture conditions, analyses, and experimental times. Disagreements were resolved by discussion and where resolution was not possible, a third reviewer was consulted (M.F.). We did not contact any study authors.

### 4.3. Methodologic Quality Assessment and Risk of Bias

In this systematic review, quality assessment of the in vitrostudies was not performed since there is no validated grading scale. In all in vivo studies, quality assessment was performed according to the Animals in Research Reporting In Vivo Experiments (ARRIVE) guidelines [55]. They consist of a 20-item checklist including all details for a minimum quality in reporting researches performed on animals. 

Riskof bias in all in vivo studies was assessed using the SYRCLE tool for animal studies that consists of a 10-item checklist [56].Overall, studies were considered at low risk of bias if all criteria were satisfied, at unclear risk of bias if one or more criteria were partly satisfied, or at high risk of bias if one or more criteria were not met. Quality criteria and risk of bias assessments were performed by two independent authors (D.C. and M.T.). Any disagreement was resolved by consensus with a third reviewer (M.F.).

## 5. Conclusions

In conclusion, there is a growing body of evidence suggesting that sex differences exist in clinical and preclinical manifestations, symptoms, and OA progression. However, at present the conflicting results obtained in animal models show several limits because of the variety of OA animal models, and the difficulty in identifying the appropriate model [54]. At the molecular level, the available data reviewed in this paper, only seem to indicate that inflammation is higher in female than males, with poor information concerning specific molecules and targets and signaling pathways involved in OA pathogenesis and progression. These data need to be highly implemented. Further challenges need to be addressed, such as the major inclusion of sex in preclinical studies, the use of advanced in vitro models and, in the era of big data, innovative technological and methodological approaches able to profile patients’ genome, transcriptome, proteome, and metabolome through multiomics analyses [57,58]. All of these approaches might help to deeply understand molecular mechanisms involved in OA development and progression, to identify key molecular players in the disease and to clarify any unrevealed male and female difference, with the final aim to characterize patient profiles and achieve a fully personalized gender-based medicine [59].

## Figures and Tables

**Table 1:** Summary of preclinical in vitro studies.

Cell Source	Cell Phenotype	OA Diagnosis	Culture Conditions	Analysis and Experimental Times	Main Results	References
Cartilage and synovial fluid from 372 late stage OA patients (64% were female) undergoing TKA	Chondrogenic progenitor cells	The patients met the ACR classification criteriafor knee OA	50,000 cells into 3D alginate bead with human fibronectin or bovine Col IV or Col II or Matrigel with or without E2 or testosterone supplementation	Measurement in synovial fluids (E2 and testosterone). IHC and immunoblotting (ERα, ERβ, and AR). Microarray analysis and RT-qPCR (ESR1, ESR2, AR, Sox9, Col I, Col II, and Runx2). FACS analyses	↑testosterone in male synovial fluid vs. female. Microarrays show that 4.9% genes exhibited expression activities different between sexes. ↓ESR1 and ESR2 after testosterone in females. ↑ESR1 and ESR2 after testosterone in males. ↑AR after E2 and testosterone in males. ↑Sox9 and Col I after E2 in females and after testosterone in males. ↓Col II after E2 and testosterone in males. ↓Runx2 after E2 in males	Koelling et al. 2010 [25]
Osteochondral tissue, meniscus, synovial membrane, and synovial fluid from 20 patients (10 males and 10 females) undergoing TKA	Chondrocytes (from 6 males and 6 females) and osteoblasts	Radiographic diagnosis (Kellgren–Lawrence grading scale)	Chondrocytes and osteoblasts were harvested from both minimally or maximally eroded zones with and without 1α,25(OH)2D3 or E2	Histology on meniscus, synovium, bone, and cartilage. Measurements on synovial fluid (1α,25(OH)2D3, MMPs, sGAGs, TGF-β1,TGF-β2, and TGF-β3). RT-qPCR on cultures (VDR, PDIA3, ESR1, ERα36, IL1A, IL1B, IL6, IL8, IL10, WNT3A, WNT5A, CTNNB, DKK1, DKK2, ACAN, Col II, and COMP) (12 h). ALP in chondrocyte culture and OC and OP in osteoblast culture (24 h)	↓pain thresholds in females at baseline. In synovial fluid: ↑MMP-1, MMP-7, MMP-9, MMP-13, HGF, SCF, SCGF-β, sGAGs, TGF-β1 and TGF-β2 in males vs. females. ↑IL2α, IL3, IL12p40, IL16, IL18, TNF-β, TRAIL, LIF, M-CSF, MIF, GRO-α, MCP-3, and MIG in females vs. males. In chondrocyte culture: ↓VDR and PDIA3 and ↑ESR1 in females vs. males. ↑IL1A, IL6, and IL8ESR1 in females vs. males. ↑WNT5A, DKK1, and DKK2 in males vs. females. E2 induced ↑ALP, ACAN, COL2A1, and COMP in females vs. males. In osteoblast culture: ↑ESR1 and ERα36 in females vs. males. ↑VDR in males vs.females. E2 induced ↑ALP and OC and ↓OP in females vs. males. 1α,25(OH)2D3 induced ↑ALP in males vs. females	Pan et al.2016 [26]
Articular cartilage from 8 patients (3 males and 5 females) undergoing TKA	Chondrocytes	Not reported	Chondrocyte cultures with or without proliferative stimulus (FGF-2)	DNA extraction, aCGH, FISH and qRT-PCR (Col I, Col II, ACAN, and IL1-β) at day 0, 4, 14, and 19	Cell expansion and growth factor addition did not cause any autosomal imbalances or loss of DNA. Cell expansion from females did not cause sex chromosome abnormalities while in males a loss of Y-chromosome sequences was observed (aCGH and FISH). No changes in gene expression between males and females	Stumm et al. 2012 [27]
Subchondral, trabecular, and cortical bone from femoral head of 11 dogs (7 males and 4 females) undergoing THR	Osteoblasts	Clinical and radiographic diagnosis	Osteoblast culture from subchondral, trabecular and cortical bone in mineralizing medium (DMEM plus β-glycerophosphate and ascorbate)	Cell number and viability (6 days). TNAP activity (1 day). Mineralization analysis (40 days)	No differences in any of the analysis parameters irrespective of the bone types between males and female	Meeson et al. 2019 [28]
Synovial membrane of 8 male and 8 female Sprague-Dawley rats (8-weeks-old)	FLSs	TMJ-OA chemically induced by Freund’s adjuvant combined with MIA	FLSs treated with or without TNF-α for 6 h; FLSs (lower chamber) in co-culture system with macrophage cell line (NR8383, in the upper chamber)	ELISA and Western blot (MCP-1). RT-PCR (CD68, MCP-1, iNOS, and IL-1β expression). Macrophage migration assay in the co-culture system (24 h)	↑iNOS, IL-1β, MCP-1 expression, macrophages number, and migration in female FLSs vs. males. No difference in the mRNA expression of iNOS, IL-1β, MCP-1, and in the migration assay from male and female FLSs without treatment of TNF-α. ↑iNOS, IL-1β, and MCP-1 in female FLSs treated with TNF-α vs. male FLSs. Female FLSs recruited more macrophages vs. male FLSs upon stimulation with TNF-α	Xue et al. 2018 [29] (in vitro)

Abbreviations: OA, Osteoarthritis; TKA, Total knee arthroplasty; ACR, American College of Rheumatology; 3D, Three-dimensional; Col, Collagen; E2, 17β-estradiol; IHC, Immunohistochemistry; ER, Estrogen receptor; AR, androgen receptor; RT-PCR, Real-time C-reactive Protein; FACS, Fluorescence-activated cell sorting; MMPs, Matrix metalloproteinases; sGAGs, Sulphated glycosaminoglycans; TGF, Transforming growth factor; IL, Interleukin; ALP, Alkaline phosphatase; VDR, Vitamin D receptor; THR, Total hip replacement; FLSs, Fibroblast-like synoviocytes; TMJ, Temporomandibular joint; MIA, Monosodium iodoacetate; FGF-2, Fibroblast growth factor-2; DMEM, Dulbecco’s modified Eagle Medium; TNF, Tumor necrosis factor; MCP, Monocyte Chemoattractant Protein; TNAP, Tissue non-specific alkaline phosphatase; ACAN, Aggrecan; COMP, Cartilage oligomeric matrix protein; OP, Osteoprotegerin; OC, Osteocalcin; DNA, Deoxyribonucleic acid; FISH, Fluorescent in situ hybridization; aCGH, Array-based comparative genomic hybridization; ELISA, Enzyme-linked immunosorbent assay; iNOS, Inducible nitric oxide synthase; HGF, Hepatocyte growth factor; M-CSF, Macrophage colony-stimulating factor; LIF, Leukemia inhibitory factor; PDIA3, Protein disulfide isomerase A3; GRO-α, Growth-regulated oncogene α; MIG, Monokine induced by gamma interferon; TRAIL, TNF-related apoptosis-inducing ligand; MIF, Macrophage migration inhibitory factor.

**Table 2:** Summary of the preclinical in vivo studies.

Animal Species and Age	OA Model and Site	Aim	Follow-up and Evaluations	Main Results	References
18 male and 20 female STR/Ort mice (5, 10, 15, 20, and 35weeks-old)	Spontaneous OA (naturally occurring). Knee	To investigate and compared age- and sex-related BMD and bone architecture	35 weeks. μCT (cancellous and cortical BMD, trabecular BV/TV, Tb.N, Tb.Th, MV, and cBV/TV). ADITT	↑OA changes, ADITT and MV in males vs. females. ↑cancellous BDM, trabecular BV/TV, Tb.N, Tb.Th, and cBV/TV in females vs. males. =cortical BDM in males and females	Uchida et al. 2012 [39]
5 OA-prone males and 5 non-prone females STR/Ort mice (8–10weeks-old: pre-OA; 18–20weeks-old: OA onset; 40+ weeks-old: advanced OA)	Spontaneous OA (naturally occurring). Knee	To evaluate tibial bone phenotype	10, 20, and 40 weeks. μCT (trabecular bone mass, number and thickness, CSA, BMD, BV/TV, ellipticity, curvature, and shape). Gait analysis. Histological score (articular cartilage lesion severity scoreon toluidine blue-stained samples, OA severity)	↑trabecular bone mass, number and thickness, CSA, BMD, and BV/TV in females vs. males. ↑gait asymmetry, OA severity, ellipticity, tibia curvature and shape deviations in males vs. females	Javaheri et al. 2018 [33]
4 male and 7 female mice lacking Frzb gene (Frzb-/-), susceptible gene for OA (7weeks-old)	Spontaneous OA (genetic modifications). Knee	To study the effect of Frzb deletion on voluntary running wheel exercise performance and OA development	6–12 months. Histological score (OARSI score on safranin-o/hematoxylin-stained samples, OA severity: cartilage damage, synovitis, and osteophyte formation). Immunofluorescence (anti-type-I and anti-type IIa myosin antibodies: muscle fiber composition of soleus and extensor digitorumlongus)	↑running performancein females vs. males. =OA severity and muscle composition in males and females	Lories et al. 2009 [35]
28 male and 28 female mice carrying a targeted mutation in Nov (Nov^del3^-/-) that causes joint degeneration (2, 6, and 12 months-old)	Spontaneous OA (genetic modifications). Knee	To determine the effect of NOV expression on the anatomy of the knee joint	2, 6, and 12 months. X-ray (anatomy ofknee joint). Histological score (OARSI score on hematoxylin/eosin, toluidine blue, safranin o-stained samples, OA severity: osteophytes, proteoglycans, fibrillation, erosion, fibrocartilage-like tissue, subchondral sclerosis, cartilage cell density, and thickness). IHC (PCNA, PARP p85, Col I, and Col X)	At 2 months: ↑cartilage cell density in males vs. females. At 6 months: ↑Col I, Col X, and OA severity in males vs. females. ↑PARP p85 in females vs. males. ↓PCNA in females vs. males. ↓cartilage cell density in males and females. At 12 months: ↑Col I, Col X, PCNA, cartilage thickness, and OA severity (with abnormal bone, enlarged epicondyles and meniscus) in males vs. females. ↓cartilage cell density in males and females	Roddy et al. 2015 [38]
12 male and 20 female mice Atg5cKO (lacking Atg5 autophagic gene in their chondrocytes) (*n* = 6 of 2 months-old; 5 males and 10 females of 6months-old; 7 males and 10 females of 12 months-old; *n* = 8 of 1month-old and *n* = 5 of 2months-old)	Spontaneous OA (genetic modifications). Surgically-induced OA (post traumatic): partial MMT. Knee	To evaluate OA development in mice without autophagy in their chondrocytes	2, 6, and 12 months (spontaneous). 1–2 months (post traumatic). Histological score (OARSI score on safranin-O-stained samples: fibrillation, loss of proteoglycan, and cartilage degradation)	At 2 months (spontaneous): no sign of joint abnormality in males and females. At 6 months (spontaneous): first signs of fibrillation and loss of proteoglycan in males. No sign of OA development in females. At 12 months (spontaneous): substantial OA in males. First signs of cartilage degradation in females. ↑OARSI score in males vs. females. At 2 months (post-traumatic): ↑OA severity in males vs. females	Bouderlique et al. 2016 [30]
35 male (2, 1017, and 20 months-old) and 15 female (10–19 months-old) wild-type C57BL/6 mice	Surgically-induced OA: DMM. Knee	To evaluate subchondral bone plate sclerosis and articular cartilage changes	2 months. Histological scores (OARSI score on safranin-o/fast green-stained samples, OA severity: cartilage degeneration). μCT (subchondral bone plate thickness and BV/TV of osteophytes)	↑OA severity and subchondral bone plate thickening in males (12–19+ months) and in females (21 months). ↑BV/TV of osteophytes in males (19+ months) and in females (18 months). ↑OA severity and subchondral bone plate thickening in males vs. females (4.5 and 12 months). =BV/TV of osteophytes in males and females (12 months)	Huang et al. 2017 [32]
Male and female CD-1, wild-type, and ADAMTS-5KO mice (lacking Adamts5 gene) on congenic C57BL/6J background (8-12 weeks-old)	Surgically-induced OA: DMM. Knee	To characterize pain-related behavior during OA	8 weeks. Histology (Toluidine blue: cartilage damage). von Frey testing (mechanical allodynia)	CD-1 mice: =OA-like lesions and allodynia in male and female. Wild-type mice: ↑OA changes and allodynia in male vs. female. ADAMTS-5 mice: no OA changes and allodynia in males and female	Malfait et al. 2010 [36]
50 male and 50 female α1integrin-deficient BALB/c mice (13±1 weeks-old)	Surgically-induced OA: DMM. Knee	To determine metabolite profiles of serum of mice treated with EGFR inhibitor erlotinib	8 and 12 weeks. µCT (OA signs: cartilage degeneration, bone volume, and subchondral bone thickness). NMR spectroscopy and OPLS-DA statistical model (metabolic profiles: dimethyl sulfone, glutamine, serotonin, 3-hydroxyisovalerate, phenylalanine)	↓OA signs in females vs.males. ↑OPLS-DA model for females vs. males (relationship between metabolic data and erlotinib treatment). ↑influence and interconnectedness of metabolites unique to males vs. to those unique to females. Glutamine identified as a potential biomarker for resistance to EGFR tyrosine kinase inhibitors. ↑dimethyl sulfone and glutamine in males and females. ↓serotonin, 3-hydroxyisovalerate and phenylalanine in males and females. No clear association between OA and metabolite profiles. Erlotinib treatment and genotype influence metabolite profile, while DMM does not	Mickiewicz et al. 2016 [37]
12 male and 12 female C57BL/6J mice (8–10weeks-old)	Surgically-induced OA: MMT. Knee	To investigate sex effects on pain and knee damage	12 weeks. Histological score (OARSI score on toluidine blue-stained samples: cartilage damage and osteophytes), mechanical and heat sensitivity (von Frey filaments and Hargreaves test), limb use and locomotor activity	↑cartilage damage in males vs. females. =osteophyte formation in males and females. Heat sensitivity in males and females, but more delayed in females vs. males. ↑mechanical sensitivity and locomotor activity in females vs. males. ↓load on limb in males vs. females	Temp et al. 2020 [40]
17 male and 10 female C57BL/6 mice with fat-1 transgene, which convert dietary n-6 to n-3 PUFAs endogenously, are fed an n-6 PUFA enriched diet (9–14 months-old)	Metabolically-induced OA: n-6 PUFAs enriched diet. Knee	To evaluate effect of fat-1 transgene expression on inflammation and idiopathicdevelopment of OA in cartilage, bone and synovium	14 months. Serum analyses: GC-FID (PUFAs) and ELISA (IL6 and TNF-α). μCT (subchondral cortical and trabecular bone: thickness, mineral density, bone spacing and BV/TV). Histological score (Mankin score, OA severity: cartilage degeneration, osteophytes, synovial thickness and extension)	↓n-6:n-3 PUFA ratio in males vs. females. ↓IL-6 and TNF-α in males and females. =subchondral cortical and trabecular bone parameters and OA severity in males and females	Cai et al. 2014 [31]
39 male and 39 female genetically modified mice hCRP, LDLr-/- and ApoE*3Leiden.CETP, based on C57BL/6J background (8–14 weeks-old)	Metabolically-induced OA: high-caloric diet (16 or 45 kcal% energy from fat). Knee	To examine impact of metabolic dysfunctionon osteophyteformation, synovial inflammation, and cartilagedegradation	38 weeks. ELISA (metabolic parameters: bodyweight, cholesterol, glucose and insulin levels). Histological score (OARSI score on hematoxylin, fast green and safranin-o -stained samples, OA severity: cartilage degradation, osteophyte formation, and synovitis)	Metabolic dysfunction in all strains. ↑OA severity, bodyweight, glucose, and insulin levelsin males vs. female (hCRP and ApoE*3Leiden.CETP). ↑cholesterol and synovitis in females vs. males (ApoE*3Leiden.CETP)	Kozijn et al. 2018 [34]
92 male and 98 female Fischer rats (3–6 months-old, young; 20–24 months-old, aged)	Chemically-induced-OA: MIA. Knee	To investigate age and sex differences in acute and chronic pain	4 weeks. Thermal, mechanical, and nociceptive sensitivity and hyperalgesia	↓thermal threshold in females vs. males and =between young and old. =mechanical threshold and nociceptive sensitivity in males and females, and in young and old. ↑hyperalgesia old vs. young and in females vs. males	Ro et al. 2019 [41]
46 male and 28 female Sprague-Dawley rats	Chemically-induced OA: unilateral MIA. TMJ	To characterize sex differences in development of ongoing pain and central sensitization	16 days. Histological score (Mankin score on toluidine blue and hematoxylin/eosin-stained samples, OA severity: cartilage loss, subchondral bone changes, osteophyte, and synovitis). Analysis of pain (meal period, meal number, and tactile sensitivity)	=pathological scores and OA severity in males and females. ↑ongoing pain and tactile hypersensitivity in females vs. males. ↓meal period in males and females. ↑meal number in males and females	Sannajust et al. 2019 [42]
10 male and 10 female Sprague-Dawley rats (8weeks-old)	Surgically-induced OA: malocclusion created by moving the first mesial molarsand the third molars distally. TMJ	To investigate expression differences of IGF1, IGFR1 and IGFBP3 in mandibular condylar cartilage	2 and 4 weeks. Histology (hematoxylin/eosin, OA-like changes: cartilage morphology and thickness). IHC and RT-PCR (Col II, IGF1, IGFR1, and IGFBP3 expression)	↑OA-like changes in females vs. males. ↓cartilage thickness, IGF1, IGFR1, IGFBP3, and Col II in females vs. males	Yu et al. 2012 [43]
5 male and 5 female Sprague-Dawley rats (8weeks-old)	Chemically-induced OA: CFA and MIA. TMJ	To evaluate sex difference in synovial inflammation, cartilage destruction, and subchondral bone deterioration	2 weeks. Histological score (score of synovial inflammation, cartilage destruction, and subchondral bone remodeling on hematoxylin/eosin and toluidine blue-stained samples, OA severity: synovitis, cartilage damage, subchondral bone sclerosis, and deterioration). μCT (BV/TV). IHC (CD68, MCP-1, iNOS and IL-1β expression)	↑OA severity, iNOS, IL-1β, MCP-1, and CD68 expression in females vs. males. ↓BV/TV in females vs. males	Xue et al. 2018 [29] (in vivo)
5 male and 5 female Hartley guinea pigs, 10 female strain 13 guinea pigs (2months-old)	Spontaneous OA (naturally occurring). Knee	To determine the association of cartilagedegeneration with subchondral BMD and meniscal MD	12 months. Histological score (Mankin score on safranin-o/fast green-stained samples: cartilage degeneration). X-ray densitometry (subchondral BMD and meniscal MD). Atomic absorption spectrophotometry (meniscal calcium content)	↑cartilage degeneration, subchondral BMD, and meniscal MD in males vs. females. =meniscal calcium content in males and females. ↑cartilage degeneration and subchondral BMD in females strain 13 vs. females Hartley. =meniscal MD in females Hartley and strain 13	Sun et al. 2015 [44]
8 males and 9 females Yorkshire pigs (7months-old)	Surgically-induced OA: bilateral transection and bridge-enhanced ACL repair with an ECM-based bioactive scaffold using absorbable or non-absorbable sutures. Knee	To evaluate role of sex on the biomechanical outcomes of bridge-enhanced ACL repair	15 weeks. Biomechanical testing (linear stiffness, yield and maximum loads, energy to failure, and knee laxity)	Absorbable suture: ↓linear stiffness, yield load, and maximum load in females vs. males. =energy to failure and knee laxity in males and female. Non-absorbable suture: =biomechanical outcomes in males and females	Kiapour et al. 2015 [46]
23 males and 18 females Yucatan mini-pigs (15±1months-old)	Surgically-induced OA: unilateral transection and ACL reconstruction using bone-patellar tendon-bone allografts with or without additional bio-enhancement (ECM-based scaffold loaded with autologous PRP). Knee	To evaluate sex differences in ACL reconstruction outcomes with regards to graft structural properties, knee laxity, and cartilage damage	15 weeks. Physical examination (weight and range of motion). Biomechanical testing (yield load, linear stiffness, maximum loads, and knee laxity). Histological score (Ligament Maturity Index on hematoxylin/eosin-stained samples: cellularity, collagen, and vascularity). Macroscopic score (India ink: cartilage damage)	=weight and range of motion (from pre-injury to post-injury), cellularity and collagen in males and females. ↓yield load, linear stiffness, and maximum load in females vs. males. ↑vascularity in males vs. females. ↑cartilage damage (in conventional reconstruction without collagen-platelet composite) and knee laxity in females vs. males	Kiapour et al. 2015 [45]
153 male and 153 female baboons *Papiohamadryas* ssp. (4.5–33 years-old)	Spontaneous OA (naturally occurring). Knee	To quantify occurrence and severity of OA	- Macroscopic score (modified Outerbridge score, OA severity: cartilage degradation and osteophytes)	↑development OA earlier and mild in males vs. females. ↑OA advanced and severe in females vs. males. =osteophytes in males and females	Macrini et al. 2013 [47]

Abbreviations: BMD, Bone mineral density; μCT, Micro-computed tomography; BV/TV, Trabecular bone volume; Tb.N, Trabecular number; Tb.Th, Trabecular thickness; MV, Medullary volume; cBV/TV, Cortical bone volume per total tissue volume; ADITT, Angular degree of internal tibial torsion; CSA, Cross sectional area; Frzb, Frizzled-related protein; OARSI, Osteoarthritis Research Society International; MMT, Medial meniscectomy; DMM, Destabilization of the medial meniscus; EGFR, Epidermal growth factor receptor; NMR, Nuclear magnetic resonance; OPLS-DA, Orthogonal partial least squares-discriminant analysis; PUFAs, Polyunsaturated fatty acids; n-6, Omega-6; n-3, Omega-3; GC-FID, Gas chromatography with flame-ionization detection; hCRP, Human C-reactive protein; CETP, Cholesteryl ester transfer protein; IGF1, Insulin-like growth factor-1; IGFR1, Insulin-like growth factor type 1 receptor; IGFBP3, Insulin-like growth factor binding protein-3; CFA, Complete Freund’s adjuvant; MD, Mineral density; ACL, Anterior cruciate ligament; ECM, Extracellular matrix; PRP, Platelet-rich plasma; NOV, Nephroblastoma overexpressed; PCNA, Proliferating cell nuclear antigen; ADAMTS, A disintegrin and metalloproteinase with thrombospondin motifs.

## Abbreviations

OA	Osteoarthritis
WHO	World Health Organization
IL	Interleukin
TNF	Tumor necrosis factor
MMPs	Matrix metalloproteinases
NO	Nitric oxide
PGE2	Prostaglandin E2
COX-2	Cyclooxygenase 2
TKA	Total knee arthroplasty
ACR	American College of Rheumatology
3D	Three-dimensional
Col	Collagen
E2	17β-estradiol
IHC	Immunohistochemistry
ER	Estrogen receptor
AR	Androgen receptor
FACS	Fluorescence-activated cell sorting
TGF	Transforming growth factor
FGF-2	Fibroblast growth factor-2
aCGH	Array-based comparative genomic hybridization
FISH	Fluorescent in situ hybridization
THR	Total hip replacement
DMEM	Dulbecco’s modified Eagle eedium
TNAP	Tissue non-specific alkaline phosphatase
ELISA	Enzyme-linked immunosorbent assay
MCP	Monocyte Chemoattractant Protein
RT-PCR	Real-time C-reactive Protein
TMJ	Temporomandibular joint
FLS	Fibroblast-like synoviocytes
sGAGs	Sulphated glycosaminoglycans
HGF	Hepatocyte growth factor
ALP	Alkaline phosphatase
BMD	Bone mineral density
μCT	Micro-computed tomography
BV/TV	Trabecular bone volume
Tb.N	Trabecular number
Tb.Th	Trabecular thickness
MV	Medullary volume
cBV/TV	Cortical bone volume per total tissue volume
ADITT	Angular degree of internal tibial torsion
CSA	Cross sectional area
Frzb	Frizzled-related protein
OARSI	Osteoarthritis Research Society International
MMT	Medial meniscectomy
DMM	Destabilization of the medial meniscus
EGFR	Epidermal growth factor receptor
NMR	Nuclear magnetic resonance
OPLS-DA	Orthogonal partial least squares-discriminant analysis
PUFAs	Polyunsaturated fatty acids
n-6	Omega-6
n-3	Omega-3
GC-FID	Gas chromatography with flame-ionization detection
hCRP	Human C-reactive protein
CETP	Cholesteryl ester transfer protein
MIA	Monosodium iodoacetate
IGF1	Insulin-like growth factor-1
IGFR1	Insulin-like growth factor type 1 receptor
IGFBP3	Insulin-like growth factor binding protein-3
CFA	Complete Freund’s adjuvant
iNOS	Inducible nitric oxide synthase
MD	Mineral density
ACL	Anterior cruciate ligament
ECM	Extracellular matrix
PRP	Platelet-rich plasma
PRISMA	Preferred Reporting Items for Systematic Reviews and Meta-Analyses
DNA	Deoxyribonucleic acid
OC	Osteocalcin
OP	Osteoprotegerin
ACAN	Aggrecan
VDR	Vitamin D receptor
M-CSF	Macrophage colony-stimulating factor
COMP	Cartilage oligomeric matrix protein
NSAIDs	Non-steroidal anti-inflammatory drugs
2D	Two-dimensional
MIG	Monokine induced by gamma interferon
PDIA3	Protein disulfide isomerase A3
LIF	Leukemia inhibitory factor
MIF	Macrophage migration inhibitory factor
GRO-α	Growth-regulated oncogene α
ARRIVE	Animals in Research Reporting In Vivo Experiments
SYRCLE	Systematic Review Centre for Laboratory animal Experimentation
TRAIL	TNF-related apoptosis-inducing ligand
NOV	Nephroblastoma overexpressed
PCNA	Proliferating cell nuclear antigen
ADAMTS	A disintegrin and metalloproteinase with thrombospondin motifs

## References

[1] Johnson V.L., Hunter D.J. (2014). The epidemiology of osteoarthritis. Best Pract. Res. Clin. Rheumatol..

[2] Hawker G.A. (2019). Osteoarthritis is a serious disease. Clin. Exp.Rheumatol..

[3] Reginster J.L., Arden N.K., Haugen I.K., Rannou F., Cavalier E., Bruyère O., Branco J., Chapurlat R., Collaud Basset S., Al-Daghri N.M. (2018). Guidelines for the conduct of pharmacological clinical trials in hand osteoarthritis: Consensus of a Working Group of the European Society on Clinical and Economic Aspects of Osteoporosis, Osteoarthritis and Musculoskeletal Diseases (ESCEO). Semin. Arthritis Rheum..

[4] Zhang W., Doherty M. (2006). EULAR recommendations for knee and hip osteoarthritis: A critique of the methodology. Br. J. Sports Med..

[5] Mannoni A., Briganti M.P., Di Bari M., Ferrucci L., Costanzo S., Serni U., Masotti G., Marchionni N. (2003). Epidemiological profile of symptomatic osteoarthritis in older adults: A population based study in Dicomano, Italy. Ann. Rheum. Dis..

[6] Dagenais S., Garbedian S., Wai E.K. (2009). Systematic review of the prevalence of radiographic primary hip osteoarthritis. Clin. Orthop. Relat. Res..

[7] Reijman M., Hazes J.M., Pols H.A., Bernsen R.M., Koes B.W., Bierma-Zeinstra S.M. (2005). Role of radiography in predicting progression of osteoarthritis of the hip: Prospective cohort study. BMJ.

[8] Neogi T., Zhang Y. (2013). Epidemiology of osteoarthritis. Rheum. Dis. Clin. North. Am..

[9] Loeser R.F., Goldring S.R., Scanzello C.R., Goldring M.B. (2012). Osteoarthritis: A disease of the joint as an organ. Arthritis Rheum..

[10] Liu-Bryan R., Terkeltaub R. (2015). Emerging regulators of the inflammatory process in osteoarthritis. Nat. Rev. Rheumatol..

[11] Reijman M., Pols H.A., Bergink A.P., Hazes J.M., Belo J.N., Lievense A.M., Bierma-Zeinstra S.M. (2007). Body mass index associated with onset and progression of osteoarthritis of the knee but not of the hip: The Rotterdam Study. Ann. Rheum. Dis..

[12] Maldonado M., Nam J. (2013). The role of changes in extracellular matrix of cartilage in the presence of inflammation on the pathology of osteoarthritis. Biomed. Res. Int..

[13] Goldring M.B., Otero M., Tsuchimochi K., Ijiri K., Li Y. (2008). Defining the roles of inflammatory and anabolic cytokines in cartilage metabolism. Ann. Rheum. Dis..

[14] Veronesi F., Della Bella E., Cepollaro S., Brogini S., Martini L., Fini M. (2016). Novel therapeutic targets in osteoarthritis: Narrative review on knock-out genes involved in disease development in mouse animal models. Cytotherapy.

[15] Schulze-Tanzil G. (2019). Intraarticular Ligament Degeneration Is Interrelated with Cartilage and Bone Destruction in Osteoarthritis. Cells.

[16] Braun H.J., Gold G.E. (2012). Diagnosis of osteoarthritis: Imaging. Bone.

[17] Maleki-Fischbach M., Jordan J.M. (2010). New developments in osteoarthritis. Sex differences in magnetic resonance imaging-based biomarkers and in those of joint metabolism. Arthritis Res. Ther..

[18] Kolhe R., Hunter M., Liu S., Jadeja R.N., Pundkar C., Mondal A.K., Mendhe B., Drewry M., Rojiani M.V., Liu Y. (2017). Gender-specific differential expression of exosomal miRNA in synovial fluid of patients with osteoarthritis. Sci. Rep..

[19] Phinyomark A., Osis S.T., Hettinga B.A., Kobsar D., Ferber R. (2016). Gender differences in gait kinematics for patients with knee osteoarthritis. BMC Musculoskelet. Disord..

[20] Boyan B.D., Hart D., Enoka R.M., Nicolella D.P., Resnick E., Berkley K.J., Sluka K.A., Kwoh C.K., Tosi L.L., O’Connor M.I. (2013). Hormonal modulation of connective tissue homeostasis and sex differences in risk for osteoarthritis of the knee. Biol. Sex. Differ..

[21] Ouellette E.A., Makowski A.L. (2006). How men and women are affected by osteoarthritis of the hand. Orthop. Clin. North. Am..

[22] Birchfield P.C. (2001). Osteoarthritis overview. Geriatr. Nurs..

[23] De Klerk B.M., Schiphof D., Groeneveld F.P., Koes B.W., van Osch G.J., van Meurs J.B., Bierma-Zeinstra S.M. (2009). No clear association between female hormonal aspects and osteoarthritis of the hand, hip and knee: A systematic review. Rheumatology.

[24] Van der Kraan P.M. (2017). Factors that influence outcome in experimental osteoarthritis. Osteoarthr. Cartil..

[25] Koelling S., Miosge N. (2010). Sex differences of chondrogenic progenitor cells in late stages of osteoarthritis. Arthritis Rheum..

[26] Pan Q., O’Connor M.I., Coutts R.D., Hyzy S.L., Olivares-Navarrete R., Schwartz Z., Boyan B.D. (2016). Characterization of osteoarthritic human knees indicates potential sex differences. Biol. Sex. Differ..

[27] Stumm M., Boger E., Gaissmaier C.G., Oßwald C., Blankenburg M., Wegner R.D., Mollenhauer J.A. (2012). Genomic chondrocyte culture profiling by array-CGH, interphase-FISH and RT-PCR. Osteoarthr. Cartil..

[28] Meeson R.L., Perpétuo I.P., Parsons K., Orriss I.R., Shah M., Pitsillides A.A., Doube M. (2019). The in vitro behaviour of canine osteoblasts derived from different bone types. BMC Vet. Res..

[29] Xue X.T., Zhang T., Cui S.J., He D.Q., Wang X.D., Yang R.L., Liu D.W., Liu Y., Gan Y.H., Kou X.X. (2018). Sexual dimorphism of estrogen-sensitized synoviocytes contributes to gender difference in temporomandibular joint osteoarthritis. Oral. Dis..

[30] Bouderlique T., Vuppalapati K.K., Newton P.T., Li L., Barenius B., Chagin A.S. (2016). Targeted deletion of Atg5 in chondrocytes promotes age-related osteoarthritis. Ann. Rheum. Dis..

[31] Cai A., Hutchison E., Hudson J., Kawashima Y., Komori N., Singh A., Brush R.S., Anderson R.E., Sonntag W.E., Matsumoto H. (2014). Metabolic enrichment of omega-3 polyunsaturated fatty acids does not reduce the onset of idiopathic knee osteoarthritis in mice. Osteoarthr. Cartil..

[32] Huang H., Skelly J.D., Ayers D.C., Song J. (2017). Age-dependent Changes in the Articular Cartilage and Subchondral Bone of C57BL/6 Mice after Surgical Destabilization of Medial Meniscus. Sci. Rep..

[33] Javaheri B., Razi H., Piles M., de Souza R., Chang Y.M., Maric-Mur I., Hopkinson M., Lee P.D., Pitsillides A.A. (2018). Sexually dimorphic tibia shape is linked to natural osteoarthritis in STR/Ort mice. Osteoarthr. Cartil..

[34] Kozijn A.E., Gierman L.M., van der Ham F., Mulder P., Morrison M.C., Kühnast S., van der Heijden R.A., Stavro P.M., van Koppen A., Pieterman E.J. (2018). Variable cartilage degradation in mice with diet-induced metabolic dysfunction: Food for thought. Osteoarthr. Cartil..

[35] Lories R.J., Peeters J., Szlufcik K., Hespel P., Luyten F.P. (2009). Deletion of frizzled-related protein reduces voluntary running exercise performance in mice. Osteoarthr. Cartil..

[36] Malfait A.M., Ritchie J., Gil A.S., Austin J.S., Hartke J., Qin W., Tortorella M.D., Mogil J.S. (2010). ADAMTS-5 deficient mice do not develop mechanical allodynia associated with osteoarthritis following medial meniscal destabilization. Osteoarthr. Cartil..

[37] Mickiewicz B., Shin S.Y., Pozzi A., Vogel H.J., Clark A.L. (2016). Serum Metabolite Profiles Are Altered by Erlotinib Treatment and the Integrin α1-Null Genotype but Not by Post-Traumatic Osteoarthritis. J. Proteome. Res..

[38] Roddy K.A., Boulter C.A. (2015). Targeted mutation of NOV/CCN3 in mice disrupts joint homeostasis and causes osteoarthritis-like disease. Osteoarthr. Cartil..

[39] Uchida K., Urabe K., Naruse K., Kozai Y., Onuma K., Mikuni-Takagaki Y., Kashima I., Ueno M., Sakai R., Itoman M. (2012). Differential age-related bone architecture changes between female and male STR/Ort mice. Exp. Anim..

[40] Temp J., Labuz D., Negrete R., Sunkara V., Machelska H. (2020). Pain and knee damage in male and female mice inthe medial meniscal transection-induced osteoarthritis. Osteoarthr. Cartil..

[41] Ro J.Y., Zhang Y., Tricou C., Yang D., da Silva J.T., Zhang R. (2019). Age and Sex Differences in Acute and Osteoarthritis-Like Pain Responses in Rats. J.Gerontol. A Biol. Sci. Med. Sci..

[42] Sannajust S., Imbert I., Eaton V., Henderson T., Liaw L., May M., Barbe M.F., King T. (2019). Females have greater susceptibility to develop ongoing pain and central sensitization in a rat model of temporomandibular joint pain. Pain.

[43] Yu S., Sun L., Liu L., Jiao K., Wang M. (2012). Differential expression of IGF1, IGFR1 and IGFBP3 in mandibular condylar cartilage between male and female rats applied with malocclusion. J. Oral. Rehabil..

[44] Sun Y., Scannell B.P., Honeycutt P.R., Mauerhan D.R., Norton J., Hanley E.N. (2015). Cartilage Degeneration, Subchondral Mineral and Meniscal Mineral Densities in Hartley and Strain 13 Guinea Pigs. Open Rheumatol. J..

[45] Kiapour A.M., Fleming B.C., Proffen B.L., Murray M.M. (2015). Sex Influences the Biomechanical Outcomes of Anterior Cruciate Ligament Reconstruction in a Preclinical Large Animal Model. Am. J. Sports Med..

[46] Kiapour A.M., Fleming B.C., Murray M.M. (2015). Biomechanical Outcomes of Bridge-enhanced Anterior Cruciate Ligament Repair Are Influenced by Sex in a Preclinical Model. Clin. Orthop. Relat. Res..

[47] Macrini T.E., Coan H.B., Levine S.M., Lerma T., Saks C.D., Araujo D.J., Bredbenner T.L., Coutts R.D., Nicolella D.P., Havill L.M. (2013). Reproductive status and sex show strong effects on knee OA in a baboon model. Osteoarthr. Cartil..

[48] Henrotin Y., Lambert C., Richette P. (2014). Importance of synovitis in osteoarthritis: Evidence for the use of glycosaminoglycans against synovial inflammation. Semin. Arthritis Rheum..

[49] Migliore A., Procopio S. (2015). Effectiveness and utility of hyaluronic acid in osteoarthritis. Clin. Cases Miner. Bone Metab..

[50] Thysen S., Luyten F.P., Lories R.J.U. (2015). Targets, models and challenges in osteoarthritis research. Dis. Models Mech..

[51] Maglio M., Brogini S., Pagani S., Giavaresi G., Tschon M. (2019). Current Trends in the Evaluation of Osteochondral Lesion Treatments: Histology, Histomorphometry, and Biomechanics in Preclinical Models. Biomed. Res. Int..

[52] Vincent T.L., Williams R.O., Maciewicz R., Silman A., Garside P. (2012). Arthritis Research UK animal models working group. Mapping pathogenesis of arthritis through small animal models. Rheumatology.

[53] Kuyinu E.L., Narayanan G., Nair L.S., Laurencin C.T. (2016). Animal models of osteoarthritis: Classification, update, and measurement of outcomes. J. Orthop. Surg. Res..

[54] Seifirad S., Haghpanah V. (2019). Inappropriate modeling of chronic and complex disorders: How to reconsider the approach in the context of predictive, preventive and personalized medicine, and translational medicine. EPMA J..

[55] Kilkenny C., Browne W.J., Cuthill I.C., Emerson M., Altman D.G. (2013). Improving bioscience research reporting: The ARRIVE guidelines for reporting animal research. Animals.

[56] Hooijmans C.R., Rovers M.M., de Vries R.B., Leenaars M., Ritskes-Hoitinga M., Langendam M.W. (2014). SYRCLE’s risk of bias tool for animal studies. BMC Med. Res. Methodol..

[57] Qian S., Golubnitschaja O., Zhan X. (2019). Chronic inflammation: Key player and biomarker-set to predict and prevent cancer development and progression based on individualized patient profiles. EPMA J..

[58] Maturo M.G., Soligo M., Gibson G., Manni L., Nardini C. (2019). The greater inflammatory pathway-high clinical potential by innovative predictive, preventive, and personalized medical approach. EPMA J..

[59] Gemmati D., Varani K., Bramanti B., Piva R., Bonaccorsi G., Trentini A., Manfrinato M.C., Tisato V., Carè A., Bellini T. (2019). "Bridging the Gap" Everything that Could Have Been Avoided If We Had Applied Gender Medicine, Pharmacogenetics and Personalized Medicine in the Gender-Omics and Sex-Omics Era. Int. J. Mol. Sci..

